# Prevalence and characteristics of patients with seronegative myasthenia gravis

**DOI:** 10.1007/s00415-026-13837-7

**Published:** 2026-05-09

**Authors:** Rebecca Kjær Andersen, Peter Ankjær, Kasper Holst Axelsen, Linda Kahr Andersen, John Vissing, Nanna Witting

**Affiliations:** Copenhagen Neuromuscular Center, Rigshospitalet, Inge Lehmanns Vej 8, Copenhagen, Denmark

**Keywords:** Myasthenia gravis, Seronegative, Skeletal muscle, Neuromuscular junction, Retrospective

## Abstract

Myasthenia gravis is an autoimmune disorder characterized by antibodies targeting the neuromuscular junction, causing fatigue of skeletal muscle. Natural history and epidemiological studies report that around 10% of patients diagnosed with myasthenia gravis do not have detectable levels of antibodies, categorized as seronegative. Theories of the pathophysiology in these patients are predominantly centered around undetectable antibody titers and antibodies against yet undefined antigens in the neuromuscular junction. This study aims to investigate the regional prevalence and characteristics of patients with seronegative myasthenia gravis. This study is a retrospective chart review of 350 patients with myasthenia gravis treated at the Copenhagen Neuromuscular Center. Of these, 15 (4.3%) were seronegative and 335 (95.7%) were seropositive. No differences were found when comparing the two groups on demographics, symptom profile, comorbidities, treatment, and diagnostic delay. The frequency of seronegative myasthenia gravis patients was lower in this cohort than previously reported. Seronegative myasthenia gravis patients were essentially indistinguishable from seropositive patients on all parameters evaluated. These findings correspond well with the emerging evidence of a similar pathophysiology in the two groups.

## Introduction

Myasthenia gravis (MG) is a chronic, autoimmune disorder characterized by antibodies that damage the postsynaptic membrane of the neuromuscular junction and block proteins essential for signal transduction, resulting in weakness and fatiguability of skeletal muscle [1, 2] Ocular muscles are often affected early, producing ptosis and diplopia [3]. 15–20% of MG patients only experience ocular symptoms throughout the course of the disease, categorized as ocular MG (oMG) [1, 4]. Weakness in other skeletal muscle groups, predominantly the extremities, bulbar, and respiratory muscles, is defined as generalized MG (gMG) [3].

Historically, around 85% of MG patients have been reported to have antibodies against the acetylcholine receptor (AChR). In recent years, Muscle-Specific Kinase (MuSK) and Lipoprotein-receptor-Related Protein 4 (LRP-4) have additionally been recognized as antibody targets in MG [1, 5]. Around 10% of patients with MG have however been reported to be consistently negative for all established MG antibodies [1, 6]. These patients are categorized as triple seronegative or simply seronegative [6, 7].

Seronegative MG (SNMG) has frequently been associated with a high prevalence of oMG and generally less-severe symptoms than AChR-positive MG [7, 8]. The pathophysiology in SNMG has been discussed extensively with predominant theories centered around a combination of undetectable antibody titers and yet undetermined antibody targets [9–11]. Antibodies against other intracellular and extracellular antigens, such as agrin and titin, have been identified in seronegative as well as seropositive MG patients, and while their prognostic and diagnostic value is recognized, their role in the pathophysiology is not clear [9, 12].

Therapeutic strategies for MG patients include acetylcholine esterase inhibitors and immunosuppressive agents. In severe cases, intravenous immunoglobulin therapy (IVIG) and plasma exchange (PLEX) can be necessary add-on therapies [13]. Thymectomy can in some cases significantly decrease symptom severity and is especially recommended in cases of early-onset AChR-positive MG and MG patients with thymoma [13, 14].

The object of this study was to investigate the prevalence of SNMG in MG patients treated at the Copenhagen Neuromuscular Center and assess whether seronegative patients differ from seropositive, in ways that might have diagnostic and therapeutic implications.

## Materials and methods

### Study design

The study is a retrospective chart review based on medical records of patients diagnosed with MG treated at the Copenhagen Neuromuscular Center. The primary outcome was antibody status. Secondary outcome measures were demographics, symptom profile, comorbidities, treatment, and diagnostic process. Data were collected from the digital medical record system used at all hospitals in the Capital and Zealand Regions of Denmark. This record system contains notes and test results dating back to 2008. In the case of patients diagnosed prior to 2008, archived documents were retrieved.

AChR and MuSK antibody tests ordered in these regions of Denmark were analyzed with radioimmunoassays. LRP-4 antibody tests were analyzed with cell-based assays.

### Subjects

Inclusion criteria were written consent from the patient and a confirmed diagnosis of MG. At least two of the following three criteria were required for a confirmed MG diagnosis: (1) a clear, immediate response to an acetylcholine esterase inhibitor agent administered either intravenously or orally, (2) transmission abnormalities detected by single fiber electromyography and/or decrement on repeated nerve stimulation or (3) detection of MG-specific antibodies. All included seronegative patients had a confirmed diagnosis based on response to acetylcholine esterase inhibitors and electromyographic abnormalities.

### Statistical analysis

A Fisher’s exact test was used to compare the seronegative and -positive patients on categorical parameters with one or more expected values < 5 or with more than two categories. Regarding parameters with two categories and all expected values > 5, a Pearson’s chi-squared test was used.

Welch’s two-sample t-test was used to compare the two groups on continuous parameters. Results are presented as mean ± standard deviation (SD). p-values < 0.05 were considered significant.

## Results

**Fig. 1:**
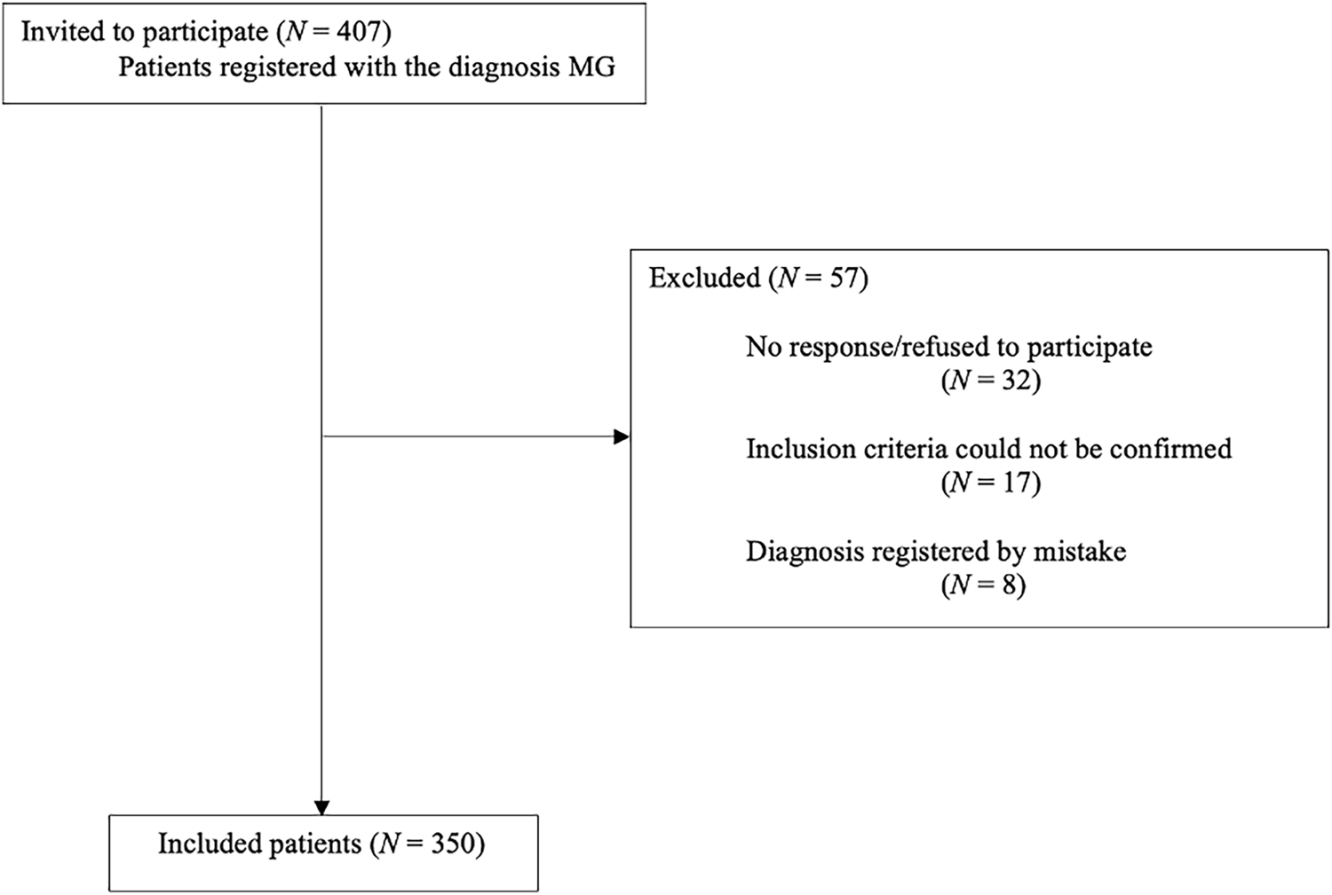
Participant inclusion process

407 patients registered with MG were invited to participate in the study. Of these 57 were excluded or did not respond (Fig. 1). 17 patients were excluded because the MG diagnosis could not be confirmed. Two of these patients were seronegative, and in one case, there was no information about antibody status. Of the 350 included patients, 15 were seronegative. 14 of the 15 seronegative patients had been tested for AChR, MuSK, and LRP-4 antibodies. One patient had only been tested for AChR antibodies. All but 3 of the seronegative patients were retested for antibody status. The seropositive patients included 326 (97,3%) AChR-AB-positive, 6 (1,8%) MuSK-AB-positive and 3 (0,9%) LRP-4-positive patients. 13 of the seronegative patients had been tested within three years of data collection.

Participant characteristics are presented in Table 1. No differences were found between the seronegative and -positive patient groups regarding age at disease onset, sex, fraction of patients with oMG, and fraction of patients with hyperplasia or thymoma. Although not statistically significant, a tendency was found for a lower prevalence of autoimmune comorbidity in the seronegative patients.

**Table 1:** Patient characteristics

	Seronegative	Seropositive	*p* value
*N*	*15 (4.3%)*	*335 (95.7%)*	
Characteristics			
Age at disease onset			
*Mean* ± *SD, years*	48 ± 17	52 ± 21	0.40
Female sex – no. (%)	8 (53)	179 (53)	1.00
Ocular symptom profile – no. (%)	3 (20)	52 (16)	0.71
Enlarged thymus – no. (%)	1 (7)	90 (27)	0.53
Thymoma	0	42	
Thymus hyperplasia	1	41	
No information on histology	0	7	
Autoimmune comorbidities – no. (%)	1 (7)	89 (27)	0.13

Indicated p-values are outcomes of statistical analysis comparing the seronegative and seropositive patient groups on each parameter

**Fig. 2:**
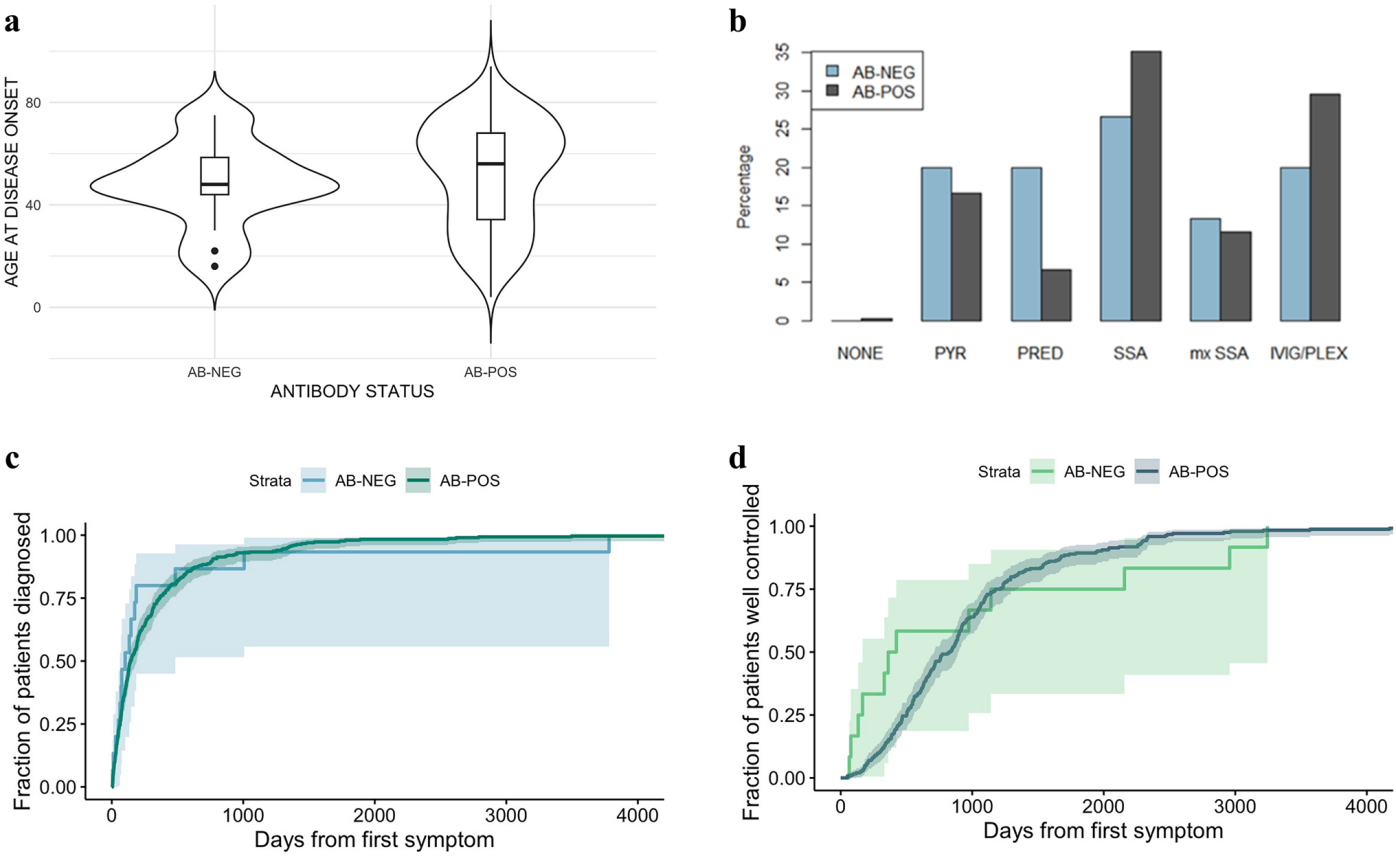
**a** Violin plot of age at disease onset in the seronegative and seropositive groups, respectively. **b** Percentage of seronegative (blue) and seropositive (black) patients in each medication category. Patients were grouped into 6 treatment intensity groups, with treatment intensity increasing, starting with NONE = no medication, PYR = treatment with pyridostigmine only, PRED = treatment with pyridostigmine and/or prednisolone, SSA = treatment with one steroid-sparing agent, mx SSA = treatment with two or more different steroid-sparing agents, and ending with IVIG/PLEX = treatment with intravenous immunoglobulin therapy or plasma exchange. **c.** Cumulative incidence plot of days from first symptom experienced to the time of confirmed diagnosis. **d.** Cumulative incidence plot of days from the first symptom to a well-controlled disease stage

The mean age at disease onset was 48 ± 17 in the seronegative group and 52 ± 21 in the seropositive group. The age distribution is illustrated in Fig. 2A, which shows a somewhat bimodal distribution in both groups.

The distribution of patients in medication intensity categories (*p* = 0.35) is illustrated in Fig. 2b. While the difference in medication intensity between the two groups was not statistically significant, a high fraction of the seropositive patients are represented in the categories of higher intensity, whereas the seronegative patients are distributed more evenly.

Figure 2c illustrates the cumulative number of days from the patient experiencing the first symptom to the point of diagnosis confirmation in each group, and Fig. 2d illustrates the cumulative number of days to reach a well-controlled disease. A well-controlled disease stage was defined by stabilization in medication dose. There was no difference in diagnostic delay (*p* = 0.71) or in time reaching a well-controlled disease stage (*p* = 0.92) between the two groups.

20 patients among the seropositive originally tested negative for antibodies, but when retested were found positive in a span of 10–3911 days after the first negative test.

## Discussion

A considerably lower fraction of seronegative patients was found in this cohort than was reported in previous studies. The number of seropositive patients initially testing negative for antibodies suggests that repeated and improved testing methods over time reduce the number of seronegative patients.

Gilhus et al. suggest that seronegative MG patients constitute a heterogeneous group consisting of patients with low-affinity antibodies or antibodies in undetectable concentrations, patients with pathologic antibodies against yet undefined antigens, and finally some patients suffering from myasthenic syndromes not mediated by antibodies [1].

On all parameters evaluated, we concluded that the seronegative patients in this cohort did not differ from the seropositive. While not finding significant differences in treatment between the two groups, we did find that a higher percentage of seronegative patients could manage their symptoms with pyridostigmine and prednisolone alone, without requiring treatment with steroid-sparing agents or IVIG/PLEX. These findings correspond well with emerging evidence that a majority of SNMG patients have circulating AChR antibodies with characteristics or at levels that require particularly sensitive assays to be detected. Leite et al. concluded that in 2/3 of patients testing negative by use of standard assays, IgG1 antibodies can be detected using cell-based assays with clustered AChRs [15]. Rodríguez Cruz et al. also found that cell-based assays with clustered AChRs are useful in diagnosing seronegative MG and furthermore concluded that patients with antibodies against clustered AChRs only tend to have a milder phenotype and an earlier onset of disease [16].

Antibodies play a key role in the diagnostic process of MG. Seronegative patients are consequently by definition more challenging to diagnose[17], and it could be speculated that some of the SNMG patients included in the study do not, in fact, have MG. We have, however, applied strict diagnostic criteria with both a response to an acetylcholinesterase inhibitor agent and transmission abnormalities by single fiber electromyography and/or decrement on repeated nerve stimulation required for SNMG diagnosis.

Due to the strict diagnostic criteria, atypical patients lacking pyridostigmine response may be missed. However, among the 17 patients excluded because MG status could not be confirmed, only 3 were either antibody negative or lacked information about antibody status.

Because of the more challenging diagnostic process for seronegative MG patients, we expected SNMG patients to have a more extensive delay in time from symptomatic debut to diagnostic confirmation, and subsequently reach a well-controlled disease stage. This was surprisingly not reflected in our results. The diagnostic delay is, however, substantial in both groups, and the implementation of cell-based assays might, in future, not only significantly reduce the number of seronegative patients, but also be a valuable factor in improving the efficiency of the diagnostic process.

### Conclusion

In conclusion, 4.3% of the 350 patients in this cohort were seronegative. No differences were found between seronegative and -positive patients when comparing the two groups on demographics, symptom profile, treatment, and diagnostic process, suggesting a common pathophysiology in the two groups. The data indicate that seronegative MG patients should be managed similarly to seropositive patients.

## Data Availability

The data that support the findings of this study are available from the corresponding author upon reasonable request.
